# Impact of Preparation of Titanium Alloys on Their Abrasive Water Jet Machining

**DOI:** 10.3390/ma14247768

**Published:** 2021-12-16

**Authors:** Adam Štefek, Martin Tyč

**Affiliations:** Department of Physics, Faculty of Electrical Engineering and Computer Science, VSB–Technical University of Ostrava, 17. listopadu 2172/15, 70800 Ostrava, Poruba, Czech Republic; martin.tyc.st@vsb.cz

**Keywords:** abrasive water jet, cutting, declination angle, traverse speed, sample preparation

## Abstract

Several titanium alloys, i.e., grade 2 Ti, Ti6Al4V and NiTi alloy, prepared by selected deformation procedures were subjected to abrasive water jet (AWJ) cutting and subsequently analysed. The study describes samples’ preparations and respective material structures. The impact of deformation processing of the selected alloys on the declination angle during cutting, and the results of measurements of surface wall quality performed for the selected samples at the Department of Physics of Faculty of Electrical Engineering and Computer Science at VŠB–Technical University of Ostrava, are presented and discussed, as are also the influences of structural features of the processed titanium alloys on surface qualities of the investigated samples. The results showed that the highest resistance to AWJ machining exhibited the Ti6Al4V alloy prepared by forward extrusion. Its declination angle (recalculated to the thickness 10 mm to compare all the studied samples) was 12.33° at the traverse speed of 100 mm/min, pumping pressure of 380 MPa, and abrasive mass flow rate of 250 g/min.

## 1. Introduction

Titanium is one of the high-tech materials featuring high corrosion resistance, low density and elasticity modulus, low thermal conductivity and high strength [1]. Ti and its alloys are widespread throughout many industrial and commercial branches, as well as in medicine [2,3,4]. For example, due to the high corrosion resistance and low thermal conductivity, Ti has promising applicability in the aerospace industry as it can be used to protect plastic composite cores from heat and moisture [5]. The same favourable properties also predetermine the usage of this material in dentistry and orthopaedics [6]. Nevertheless, the advantageous behaviour of products made of Ti alloys can be supported by optimised processing of the alloys via plastic deformation, which can favourably affect the structures and consequently the properties of Ti -based materials [7,8,9].

Many experiments involving severely deformed and thermomechanically processed Ti-based alloys were performed in the past. For example, the severe plastic deformation (SPD) method of equal channel angular pressing (ECAP) was used to alter the properties of CP (commercial purity) Ti [10,11,12], as well as to manufacture ultra-fine grained samples from Ti6Al4V [13,14], NiTi alloys [15,16] or various biocompatible Ti-based alloys [17,18]. The influence of the aging of titanium alloys at a certain temperature was well described by Gupta et al. [19,20]. Samples of titanium alloy Ti-5Al-3Mo-1.5V were forged at certain temperature and deformed and subsequently heat treated in three different ways. The effect of aging of these samples was then examined. With increasing aging time (8 h) and higher aging temperature (540 °C) there was an increase in strength, hardness, but at the same time a decrease in the maximum elongation of the sample. This was caused by the formation of a secondary alpha phase that acted as a precipitate [19]. 

Due to the fact that the final products made from the deformation processed materials have to be finished by machining, researchers have also devoted their efforts to finding optimised machining processes and determining the ideal machining parameters for Ti-based materials.

The growing need to machine Ti-based alloys, widely used in almost all branches of industry, to produce components with high quality and precision, leads to the research and development of alternative machining tools. Some kinds of modern materials, including Ti-based alloys, are hard to be machined by conventional methods, due to overheating and quick blunting of tools, its clogging or destruction, which can be caused by high ductility of the machined material, its low thermal conductivity, etc. Therefore, unconventional machining methods can be suitable alternatives, although, given by their flexibility (non-stiffness), their precision can be lower than that of the classical tools. 

Despite the fact that many new machining methods, such as laser beams or electro-erosive processes, have been studied, one of the most used and investigated machining tools is the abrasive water jet (AWJ). The research of this tool has been carried on for years and is still ongoing [21,22]. AWJ is a universal tool that has been used for more than 40 years to machine various materials. It has been tested namely for cutting [23], milling [24,25,26,27] and peening [28,29]. However, cutting is the most developed application of AWJ. The main purpose of the majority of published studies was to predict the average depth of cut of the jet, and the final quality of the surface subjected to milling. A thorough description of AWJ machining was performed by Hashish [30,31], who adopted previous models of removing the material in deformation mode (published by Bitter [32,33]), and removing the material in cutting mode (studied by Sheldon and Finnie [34,35]). Later on, some further models based either on a large amount of experimental work [36,37], or on theoretical descriptions of the processes [38,39], were presented. Several statistical models were also presented [40,41,42,43], but their validity on materials studied under experimental conditions is limited, as demonstrated e.g., in [44]. 

**Figure 1:**
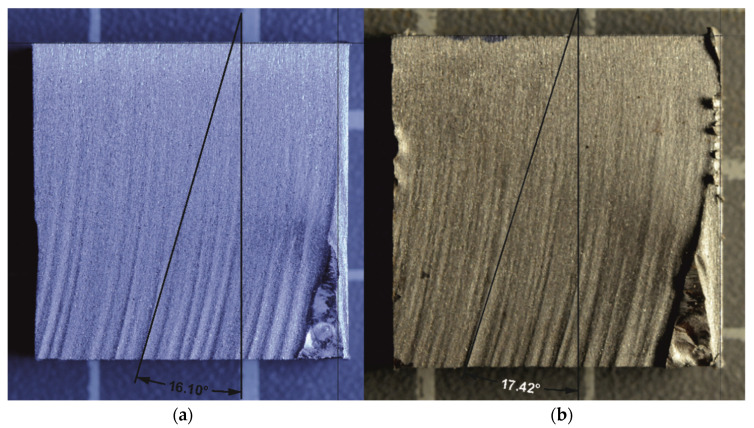
The declination angle measurement for titanium samples with traverse speed of 60 mm/min (**a**) and 75 mm/min (**b**).

Models of the interactions of AWJ with specimens’ surfaces are of great importance. Some of them consider material parameters, such as Young modulus of elasticity, grain size, material strength and density, while others are based on the previous determination of experimental values. However, most of the models can be used solely for brittle materials [45,46,47], or ductile ones [48,49,50]. The models can only rarely be applied for both the material groups. These are namely the analytical models, e.g., model derived from physical analysis of the process [39]. Nevertheless, the application of such models still requires initial experimental testing to be performed on the investigated material to determine its response to AWJ. Therefore, the analytical models are not commercially used. The description of jet trajectory and its curvature is an essential part of all the used models. Hlaváč [38] defined the curvature of the trajectory within the material through the declination angle. Figure 1 shows a comparison of the declination angle measurement of the two samples with different traverse speed set up. It is clearly visible that with increasing depth of cut, the quality of the wall surface deteriorates. Moreover, the curvature (declination angle) of the jet increases and therefore it is possible to relate the quality of the wall with the declination angle. This phenomenon is a consequence of the decreasing energy of the jet in a material. The declination angle can also be used to determine the parameter of machinability [39]. Based on these facts, information about the machinability of the respective material can be acquired just via performing a single experimental cut unlike complicated calculations via completely theoretical model. Moreover, some of the constants in the theoretical model are hard to determine and it is more time consuming than the above-mentioned semi-experimental method.

The conclusions presented in the study [39] were considered during determining the cutting parameters in this presented investigation.

All the referenced works point to the importance of further studying AWJ machining of titanium and its alloys, namely Ti6Al4V. The presented study is aimed at determination of machinability of selected Ti-based materials, it primarily characterises the effects of material structures imparted by the intensive plastic deformation-based preparation processes on the AWJ machining—cutting process. Evaluation of the machinability was performed based on the declination angle.

## 2. Experimental Setup

**Figure 2:**
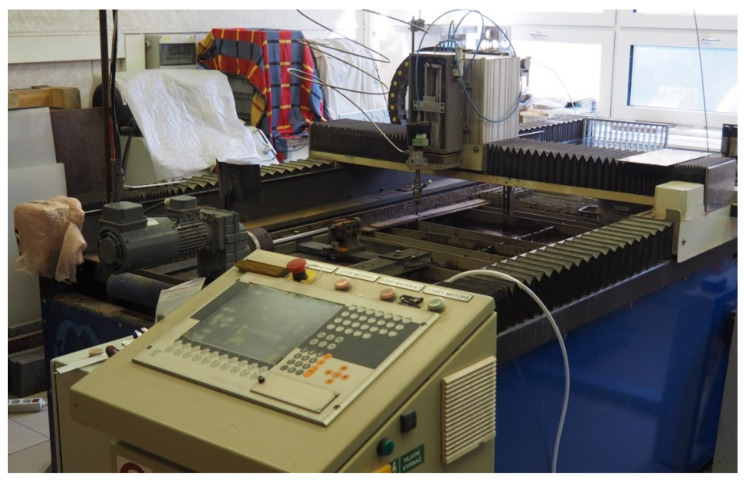
Abrasive waterjet laboratory with PTV WJ1020-1Z-EKO device.

The experiments were performed in the Laboratory of Liquid Jet at the Faculty of Electrical Engineering and Computer Science at VŠB–Technical University of Ostrava. An AWJ x-y cutting table with manually driven z-axis PTV WJ1020-1Z-EKO (PTV s.r.o., Hostivice, Czech Republic) was used (Figure 2).

Selected AWJ parameters were constant during the experiments (summarised in Table 1).

For the AWJ experiments, samples from six individual Ti-based materials manufactured under various thermomechanical conditions affecting the materials’ structures were prepared. Those were subsequently cut via the water jet and the respective declination angles on the kerf walls were measured. Summary of the samples taken from the selected materials manufactured at the Faculty of Materials Science and Technology, VŠB–Technical University of Ostrava, via vacuum induction melting (VIM), and subsequent processing via equal channel angular pressing (ECAP), forward extrusion, or rotary swaging, is given in Table 2. Sample number 5a was not cut with AWJ. However, the microstructure analysis of this sample was performed for comparison with sample 5b, which was rotary swaged.

**Figure 3:**
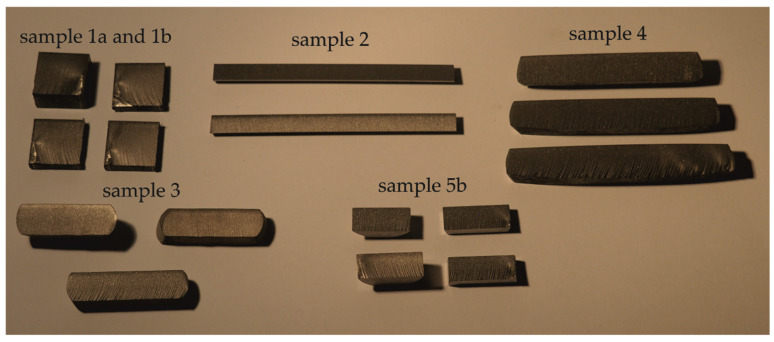
Investigated samples with different dimensions.

All of the measured samples are shown in Figure 3. 

Microstructural observations of the prepared samples were carried out using Olympus DSX1000 digital optical microscope (Olympus Corp., Tokyo, Japan), scanning electron microscope (SEM) Lyra 3 XMU (Tescan, Brno, Czech Republic). Detailed examination of the sample prepared via ECAP was performed using the transmission electron microscope (TEM) JEM-2100 (JEOL, Tokyo, Japan) operating at 200 kV. The preparation of samples taken from the selected material states was carried out via combining manual grinding on SiC papers and polishing using colloidal silica. The foil for TEM was finished by electrolytical etching.

The thicknesses of the samples and the respective traverse speeds used for cutting are listed in Table 3. The samples were left in their “natural” thicknesses produced during the preparation processes to prevent inducing possible changes during machining performed to prepare samples with identical thicknesses. Samples have been collected over the years as a by-product of previous research. Some of them were even stored for several years. The structural changes, which took place at room temperature throughout the years are marked as natural aging. As the samples were not primarily intended to measure and evaluate the declination angle, their thicknesses varied and therefore different traverse speeds were used to achieve a similar surface quality. The chosen traverse speed depended also on the material structure and composition. The traverse speed sets for each of the samples were determined according to the experimental cut and material thickness based on the model presented in [39]. The speed sets were selected so that, if possible, the declination angles varied between 10°and 30°. The selected range of speeds and relevant angles corresponded to the most common industrial requirements on quality (also considering the economics of the process). After the AWJ cuts were performed, the kerf walls were photographed and the declination angles were measured on them ten times in the CorelDRAW^®^ Home & Student 2018 software [51] according to the methodology presented first in ref. [38] and used in the Laboratory of Liquid Jet at Faculty of Electrical Engineering and Computer Science at VSB—Technical University of Ostrava.

## 3. Results 

### 3.1. Microstructural Observations

**Figure 4:**
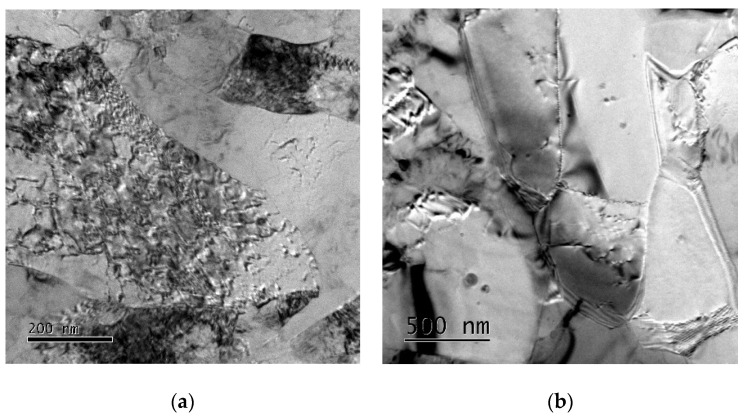
Substructures of: Sample 1a (**a**); Sample 1b (**b**).

Sample 1a was prepared from a TiCP2 10-10x120 mm billet extruded via double-pass ECAP through a die with 90° angle; the TEM image of the (sub)structure of this sample is presented in Figure 4a. It is visible that the structure of the sample contained fine grains featuring a high density of dislocations accumulating in the dislocation cells, as well as forming sub grains. These structure phenomena contributed to the increased hardness observed just after the preparation. 

The material has also been subjected to 10 years of natural aging; the substructure of Sample 1b, characterised with a large portion of dislocations-free recrystallised grains, is depicted in Figure 4b. Both the samples were taken from transversal cuts through the processed billet.

**Figure 5:**
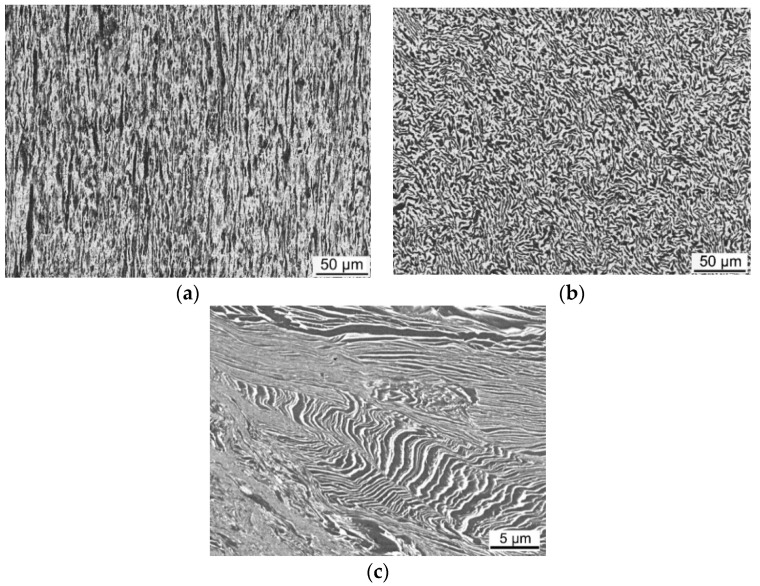
Structures of Ti6Al4V samples: sample 2—axial cut from rod subjected to hot forward extrusion (**a**); sample 3—transversal cut from rod subjected to hot forging (**b**); sample 4—transversal cut from rod subjected to three-step cold stamping (**c**).

Sample 2 was prepared from the Ti6Al4V alloy manufactured by hot forward extrusion (900 °C). The extruded rod with a diameter of 8 mm was cut along the extrusion axis to prepare the sample. The image of the structure of sample 2, taken from the axial longitudinal cut, acquired via SEM, is presented in Figure 5a. The figure shows significantly deformed alpha and beta phase grains elongated in the direction of the dominant plastic flow, i.e., in the extrusion direction.

Another Ti6Al4V sample, denoted in this work as sample 3, was hot forged at 900 °C, from a rod with the original diameter of 10 mm, with the deformation ratio of 60% (in the direction parallel to the forged rod axis). Before the examination, the material was naturally aged for 4 years. The microstructure of this sample, depicted in Figure 5b, was analysed via SEM in the direction perpendicular to the original forging axis. In other words, sample 2 was examined in the longitudinal direction of the forged rod to demonstrate the effects of forging on elongation of the grains, whereas sample 3 was examined in the transversal direction to show the effects of the deformation performed in the perpendicular direction. Figure 5b clearly depicts the deformed alpha and beta phase grains.

The last sample prepared from the Ti6Al4V alloy was sample 4, which was deformed by three consequential deformation (stamping) steps at room temperature, i.e., under cold conditions, with the total deformation ratio of 45%. Before examination, the material was again naturally aged for 4 years, similar to sample 3. The deformation was performed on the originally extruded rod in the direction parallel to the extrusion axis. The structure acquired via SEM from the transversal cut of the stamped extruded rod is depicted in Figure 5c. The image again clearly depicts deformed alpha and beta grains. The grains are evidently heavily deformed and even shear bands start to develop, since the restoration processes during the room-temperature deformation were aggravated.

**Figure 6:**
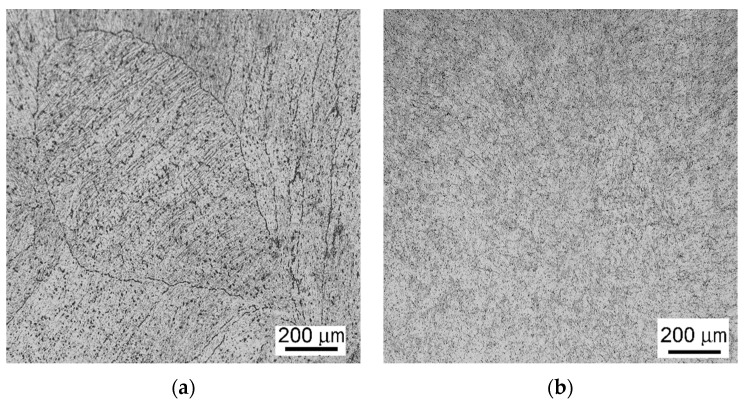
Structures of NiTi samples: sample 5a—as-cast, prepared by VIM (**a**); sample 5b—as-cast and rotary swaged (**b**).

Further, samples from a NiTi alloy, the chemical composition of which was (in wt.%) 50% Ni and 50% Ti, were prepared. This alloy was, in the first step, prepared by VIM. From the as-cast rod, a sample in the transversal direction was prepared (sample 5a, the image of the structure of which acquired via optical microscopy is depicted in Figure 6a). Subsequently, the cast rod was processed via rotary swaging with a total deformation ratio of 0.9 [52,53]. The structure image of sample 5b, taken from the swaged rod, acquired via optical microscopy is shown in Figure 6b.

Last but not least, an additional sample (sample 6) from an as-cast CPTi2 plate without any mechanical or thermomechanical treatment was prepared. The structure of this sample was not prepared, since all the cut pieces were handed over to further processing and evaluation of kerf walls immediately after cutting. The sample was selected since the age of this sample was unknown, which enables final verification of the used model on the estimation of the probable age of this sample. 

### 3.2. Declination Angles

Mean values of the declination angles were calculated for all the cuts of all the prepared samples. The results for all the samples, their respective thicknesses and traverse speeds are summarised in Table 4. Relative uncertainty for all the measurements was 1–5%.

Since the conditions of these measurements are different, the declination angle needs to be recalculated through the theoretical model to the same conditions. Therefore, this investigation builds on previous research made by Hlaváč et al. His research was focused on verification of the theoretical model describing final declination angle on two different Titanium samples [23].

## 4. Discussion

To perform a thorough study, the AWJ cuts were performed for a variety of samples with slightly different dimensions, and under numerous traverse speeds. Therefore, due to the fact that the initial conditions were different for the individual cuts, the theoretical model by Hlaváč presented in [38,39], which was used in this study, was applied after the declination angles were recalculated for the ideal thickness of 10 mm for all the samples, and the traverse speeds were unified to 100 mm/min. These recalculated values for the respective experimental results are summarised in Table 5. 

As regards the CPTi2 material, the comparison of samples 1a and 1b, prepared from a CPTi2 billet via room-temperature ECAP, and room-temperature ECAP followed by 10 years of natural aging, respectively, provides the basis to acquire the “rheology factor” for this material. The average calculated “degradation factor” for the CPTi2 billet, determined from the difference between the average declination angle values of 11.55° for the as-processed billet and 8.03° for the billet after 10 years of aging, is 0.35° per year.

This calculated “degradation factor” was further applied also during the evaluation of other samples subjected to natural aging before processing, i.e., the Ti6Al4V samples, sample 3 and sample 4. The declination angles, calculated using this factor, are also depicted in Table 5. For the last sample (sample 6), the age of which is not exactly known (according to internal records estimated to be 3 to 4 years), it is also possible to determine the probable aging time after the preparation of the sample. Both the values were calculated, i.e., for the estimated aging of 3 years and for the estimated aging of 4 years. The respective values were added into Table 5.

Comparing the declination angles recalculated for the identical material thicknesses and the identical traverse speeds, it is evident that Sample 2 made of Ti6Al4V has the lowest machinability (the highest declination angle). The lowest declination angle was acquired during cutting of Sample 1b (TiCP2), which was extruded through the ECAP matrix 10 years ago and is now evaluated. Nevertheless, Sample 1a was cut for the first time shortly after its processing. The average declination angle determined from the photo of the cut made on the originally processed material is substantially higher. Because the declination angle is closely related to the material properties of the sample, it is obvious that these properties, imparted by the original mechanical and/or thermal treatment, change in time. Therefore, it is necessary to take the time rheology into account when optimising the material treatment.

When the “rheology factor”, determined for Sample 1, is used also for other materials refined by mechanical and thermal processes, and subsequently subjected to natural aging (sample 3 and sample 4), the resulting average declination angle, the measure of the material resistance to the AWJ machining, can easily be used for comparison of materials in their original states. It is evident that the lowest average declination angle (the most favourable machinability) has the TiNi alloy (sample 5b). The highest average declination angle (the least favourable machinability) has the Ti6Al4V alloy prepared by forward hot extrusion (sample 2). It is evident that the machinability of the TiCP2 material is similar to that observed for the as-processed sample after 2 ECAP passes (sample 1a) and in the expected as-processed of the as-cast sample (sample 6). The Ti6Al4V alloy has lower average declination angles (better machinability) compared to the as-processed TiCP2, nevertheless the average declination angle is lower for the hot forged Ti6Al4V sample (sample 3), while for the three-step cold stamping Ti6Al4V sample, it is close to the as-processed TiCP2 samples.

## 5. Conclusions

The experiments performed on selected TiCP 2 and Ti-based alloys samples show that their resistance to AWJ cutting depends on the type of material, its preparation, and time of ageing. The hot forward extruded Ti6Al4V is the most resistant from the studied Ti6Al4V samples, while the hot forged Ti6Al4V seems to be the less resistant among them. However, the relative difference between these two materials is only 12.8%. The TiNi alloy is the least resistant of all the studied samples, as it was expected due to the Ni presence. The TiCP2 and the cold stamped Ti6Al4V have similar resistances because the differences are not exceeding the typical uncertainty of the selected experimental procedure and data processing (5%). The most important result seems to be the influence of material aging documented on TiCP2 sample processed by the ECAP technology—extrusion through a rectangular channel. The sample subjected to 10 years of natural aging was found to have 30% reduction in the AWJ machining resistance. Therefore, special attention must be paid to the rheology of materials treated by mechanical processes (extrusion, forging, rolling).

## Figures and Tables

**Table 1:** Setup of basic experimental variables of AWJ (fixed variables).

Pumping pressure (MPa)	380
Water orifice diameter (mm)	0.25
Focusing tube diameter (mm)	0.76
Focusing tube length (mm)	76
Stand-off distance (mm)	2
Abrasive mass flow rate (g/min)	250
Abrasive (Australian garnet) grain size	80 MESH

**Table 2:** Characterization of individual samples and their preparation methods.

Sample	Material	Manufacturing
Sample 1a	TiCP2	room-temperature two pass ECAP
Sample 1b	TiCP2	room-temperature two pass ECAP, 10 years of aging
Sample 2	Ti6Al4V	hot forward extrusion (900 °C)
Sample 3	Ti6Al4V	hot forging (900 °C, deformation ratio 60%), 4 years of aging
Sample 4	Ti6Al4V	three-step cold stamping (deformation ratio 45%), 4 years of aging
Sample 5a	50Ni-50Ti	as-cast by VIM
Sample 5b	50Ni-50Ti	rotary swaged after VIM (swaging degree 0.9)
Sample 6	TiCP2	as-cast, aging unknown

**Table 3:** Summary of traverse speed sets for examined material samples and samples’ thicknesses.

Sample	Material Thickness (mm)	Traverse Speed Sets (mm/min)
Sample 1a	20	30, 40, 60
Sample 1b	20	50, 75, 100, 125
Sample 2	8	100, 150, 200
Sample 3	12	100, 150, 200
Sample 4	12.5–13.5	100, 150, 200
Sample 5b	10	100, 150, 200
Sample 6	25	30, 60, 90

**Table 4:** Average declination angles measured for examined samples.

Sample	Thickness (mm)	Traverse Speed (mm/min)	Average Declination Angle (°)
Sample 1a	20	30	10.26
		40	12.73
		60	19.18
Sample 1b	20	50	12.46
		75	17.36
		100	21.80
		125	26.31
Sample 2	8	100	10.43
		150	12.31
		200	15.66
Sample 3	12	100	12.25
		150	17.30
		200	27.00
Sample 4	12.5	100	13.80
	13.2	150	21.52
	13.5	200	32.60
Sample 5b	10	100	11.36
		150	15.36
		200	21.66
Sample 6	25	30	10.72
		60	24.03
		90	42.52

**Table 5:** Declination angles recalculated from measured average declination angles for individual samples to values corresponding to the thickness of 10 mm and traverse speed of 100 mm/min, also including correction for aging and average angle with aging.

	MADA * (°)	RADA ** (°)	RADA ** with Aging (°)	Average RADA ** with Aging (°)
Sample 1a	10.26	12.09	12.09	
0 years	12.73	11.26	11.26	11.55
	19.18	11.30	11.30	
Sample 1b	12.46	8.81	12.32	
10 years	17.36	8.18	11.70	8.03
	21.80	7.71	11.22	
	26.31	7.44	10.96	
Sample 2	10.43	14.58	14.58	
0 years	12.31	11.46	11.46	12.33
	15.66	10.94	10.94	
Sample 3	12.25	9.32	10.72	
4 years	17.30	8.77	10.17	10.85
	27.00	10.27	11.67	
Sample 4	13.80	9.87	11.27	
4 years	21.52	9.46	10.86	11.31
	32.60	10.39	11.79	
Sample 5b	11.36	11.36	11.36	
0 years	15.36	10.24	10.24	10.81
	21.66	10.83	10.83	
Sample 6	10.72	9.04	10.09	
3 years	24.03	10.13	11.18	11.42
	42.52	11.95	13.00	
Sample 6	10.72	9.04	10.44	
4 years	24.03	10.13	11.53	11.77
	42.52	11.95	13.35	

* MADA—measured average declination angle; ** RADA—recalculated average declination angle.

## Data Availability

No public data were reported.
